# A tripartite paternally methylated region within the Gpr1-Zdbf2 imprinted domain on mouse chromosome 1 identified by meDIP-on-chip

**DOI:** 10.1093/nar/gku624

**Published:** 2014-07-12

**Authors:** H. Hiura, A. Sugawara, H. Ogawa, R.M. John, N. Miyauchi, Y. Miyanari, T. Horiike, Y. Li, N. Yaegashi, H. Sasaki, T. Kono, T. Arima

Nucl. Acids Res. (2010) 38 (15): 4929-4945. doi:10.1093/nar/gkq200

Editorial Expression of Concern The Editors express a note of concern regarding the article “A tripartite paternally methylated region within the Gpr1-Zdbf2 imprinted domain on mouse chromosome 1 identified by meDIP-on-chip “ by Hiura H, Sugawara A, Ogawa H, John RM, Miyauchi N, Miyanari Y, Horiike T, Li Y, Yaegashi N, Sasaki H, Kono T, and Arima T, which was published in issue 15, volume 38 (2010) of Nucleic Acids Research.

The Editors wish to alert the readers that questions have been raised about the validity of some of the figures presented in this article. The Editors note that Figures 2, 4, 6 and S3 contain duplicate panels. New figures are available in the Supplementary Data accompanying this expression of concern. Having reviewed the original data, we believe that the results and conclusion of the article are valid but wish to alert our readership to these concerns.

Keith Fox Barry Stoddard Senior Executive Editors Nucleic Acids Research

## Supplementary Material

SUPPLEMENTARY DATA

