# Supplementary material for: A tripartite paternally methylated region within the Gpr1-Zdbf2 imprinted domain on mouse chromosome 1 identified by meDIP-on-chip
Source: Nucleic Acids Res. 2014 Jul 12;42(16):10869. doi: 10.1093/nar/gku624 (PMC4176375; doi:10.1093/nar/gku624)
Supplement: SUPPLEMENTARY DATA [file supp_42_16_10869__index.html]

A tripartite paternally methylated region within the Gpr1-Zdbf2 imprinted domain on mouse chromosome 1 identified by meDIP-on-chip — SUPPLEMENTARY DATA 

# A tripartite paternally methylated region within the Gpr1-Zdbf2 imprinted domain on mouse chromosome 1 identified by meDIP-on-chip

## SUPPLEMENTARY DATA

**Files in this Data Supplement:**

- SUPPLEMENTARY DATA
