# Supplementary figures and images for: A tripartite paternally methylated region within the Gpr1-Zdbf2 imprinted domain on mouse chromosome 1 identified by meDIP-on-chip
Source: Nucleic Acids Res. 2014 Jul 12;42(16):10869. doi: 10.1093/nar/gku624 (PMC4176375; doi:10.1093/nar/gku624)

Figure 2-2

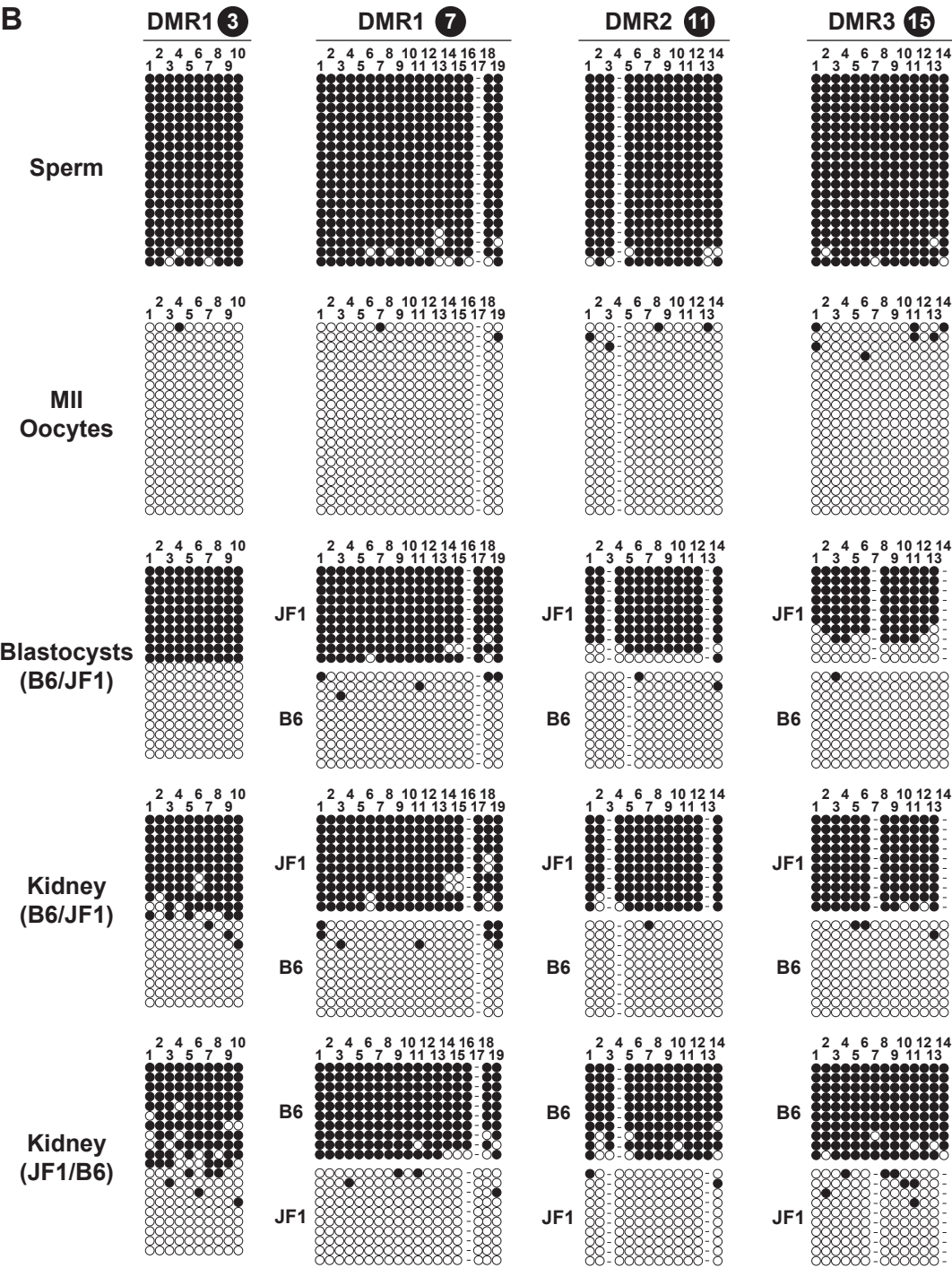

Figure 4

A *Gpr1* (NM\_146250)

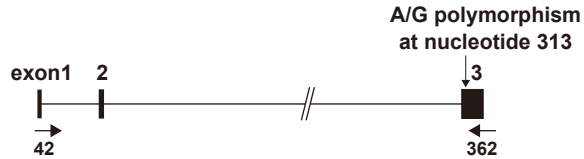

B

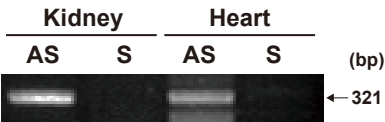

C

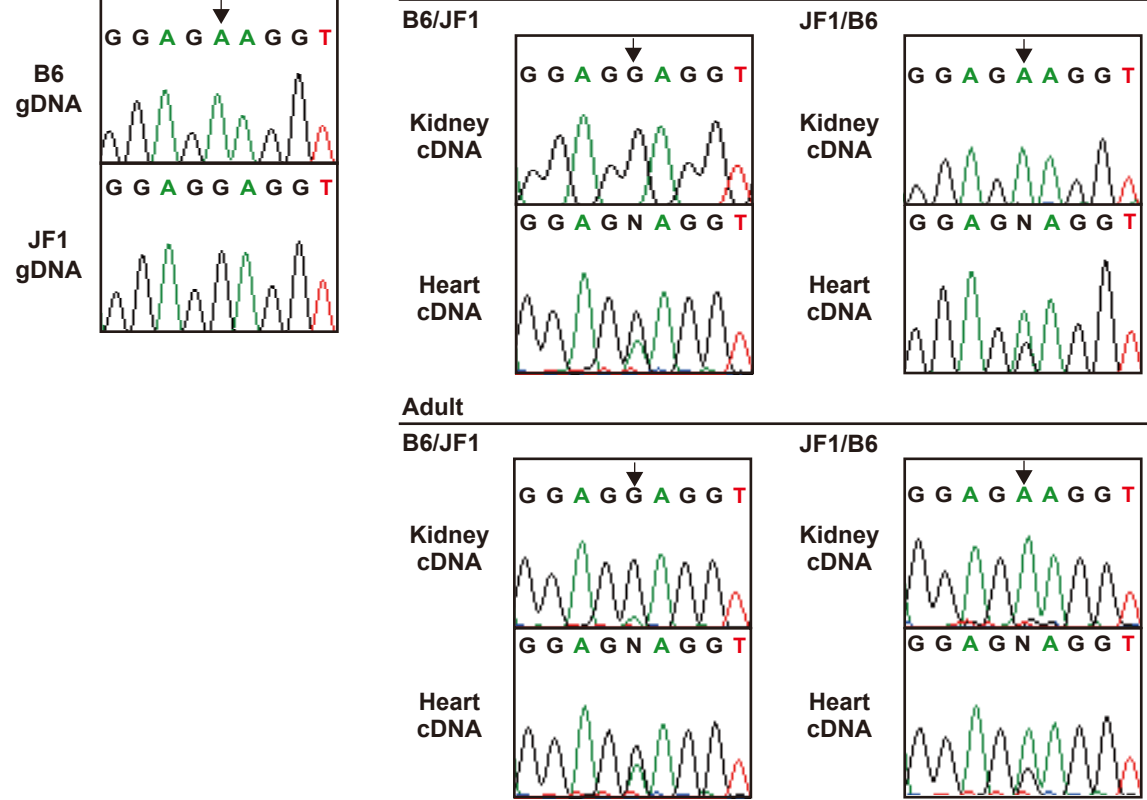

Figure 6

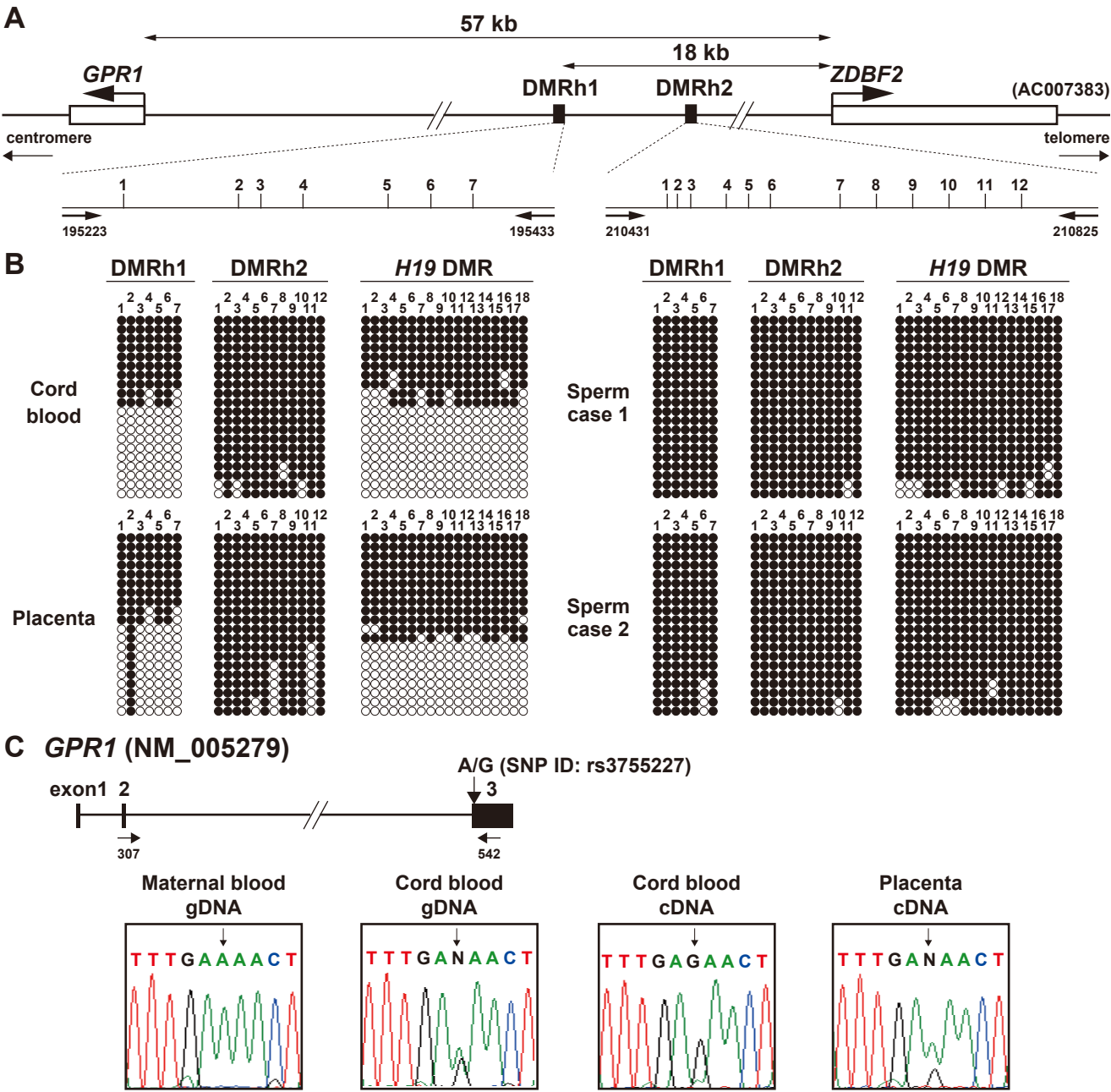

Supplemental Figure S3

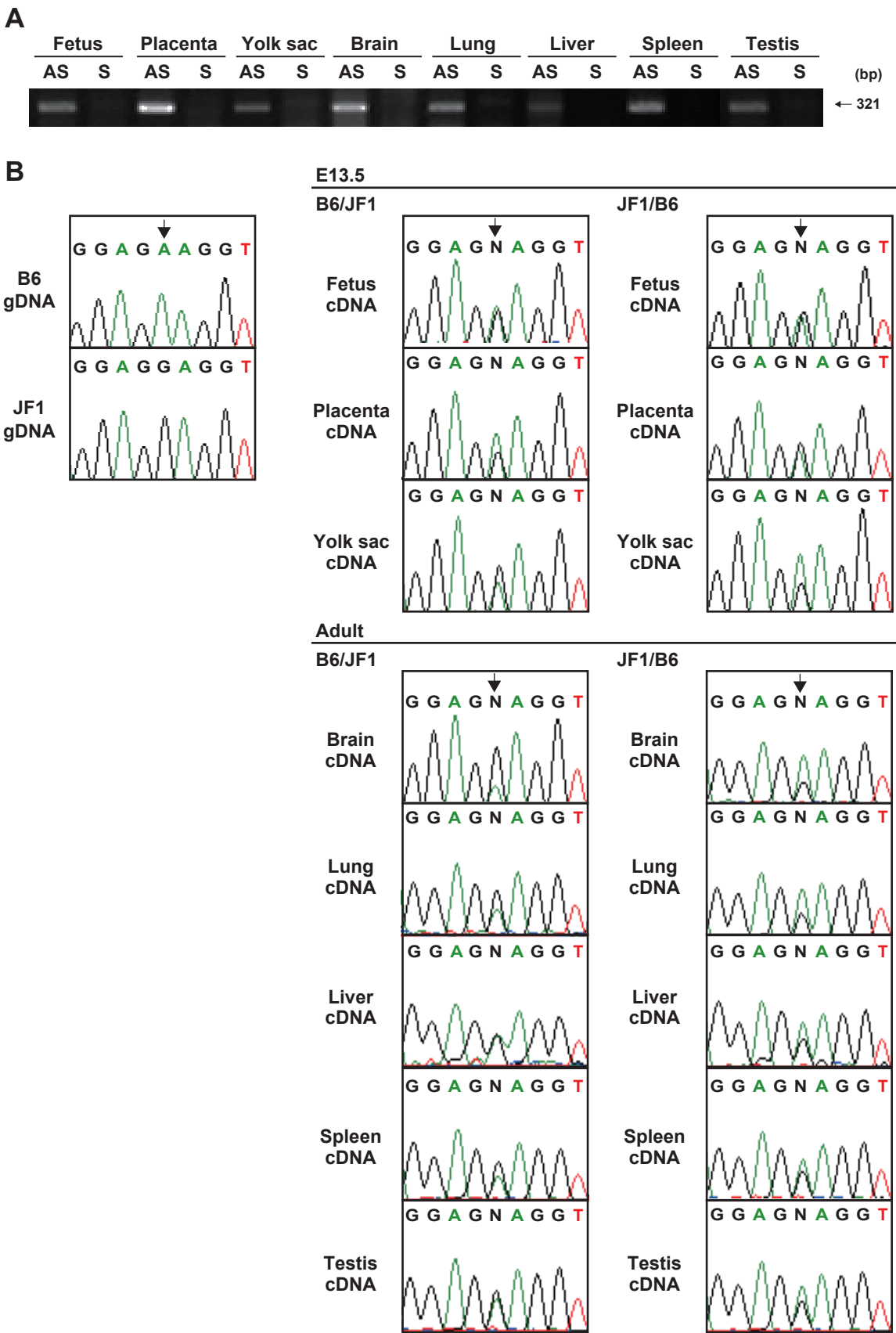

Supplement: SUPPLEMENTARY DATA [file supp_gku624_Supplementary_Data.pdf]
